# Severe pediatric Mycoplasma pneumonia as the cause of diffuse alveolar hemorrhage requiring veno-venous extracorporeal membrane oxygenation: A case report

**DOI:** 10.3389/fped.2022.925655

**Published:** 2023-01-06

**Authors:** Xinjuan Zhang, Yanping Yu

**Affiliations:** Department of Pediatric Intensive Care Unit, Affiliated to Hangzhou First People's Hospital, Zhejiang University School of Medicine, Hangzhou, China

**Keywords:** mycoplasma pneumoniae, diffuse alveolar hemorrhage, pediatric, acute respiratory distress syndrome, veno-venous extracorporeal membrane oxygenation, mNGS

## Abstract

**Background:**

Diffuse alveolar hemorrhage (DAH) is an acute life-threatening disease often associated with immunocompromised patients and systemic disorders, such as infections, vasculitis, and toxins. *Mycoplasma pneumoniae* is one of the most common causes of community-acquired pneumonia in children, which rarely causes respiratory failure and fulminant disease; However, a rapid progression may occur in some patients. Mycoplasma pneumonia-associated DAH is rare.

**Case Presentation:**

We report a case of severe pediatric mycoplasma pneumonia in an immuno-competent child. This patient's condition progressed rapidly, with severe lung lesions associated with pleural effusion, coagulopathy, diffuse alveolar haemorrhage and severe respiratory distress requiring ventilator and intravenous extracorporeal membrane oxygenation (VV-ECMO) support. She was discharged upon successful treatment.

**Conclusion:**

Diffuse alveolar hemorrhage associated with *Mycoplasma pneumoniae* in children is very rare, and clinicians should be aware of the potential rapid onset of the disease. Early detection and diagnosis are very important. The main treatment measures include anti-infection and supportive measures such as mechanical ventilation, but as in our case, success with both prone positioning for more than 10 h per day and VV-ECMO was life-saving.

## Introduction

Diffuse alveolar hemorrhage (DAH) is a life-threatening emergency, and hospital mortality rates are reported to be 20%–100% (1). Its etiologies include vasculitis, thrombocytopenia, autoimmune diseases, coagulopathy, drugs, and infections (2). *Mycoplasma pneumoniae* (MP) is one of the most common causative organisms of community-acquired pneumonia in children. It rarely causes life-threatening disease, but some patients can develop a rapid progression of MP-associated disease. MP-associated DAH is very rare and has been reported to occur more frequently in immunocompromised patients (3). We report a previously healthy child who suffered from severe mycoplasma pneumonia, complicated with DAH and severe respiratory distress syndrome. The patient required ventilator and extracorporeal membrane oxygenation (ECMO) support and was treated successfully.

## Case presentation

**Figure 1:**
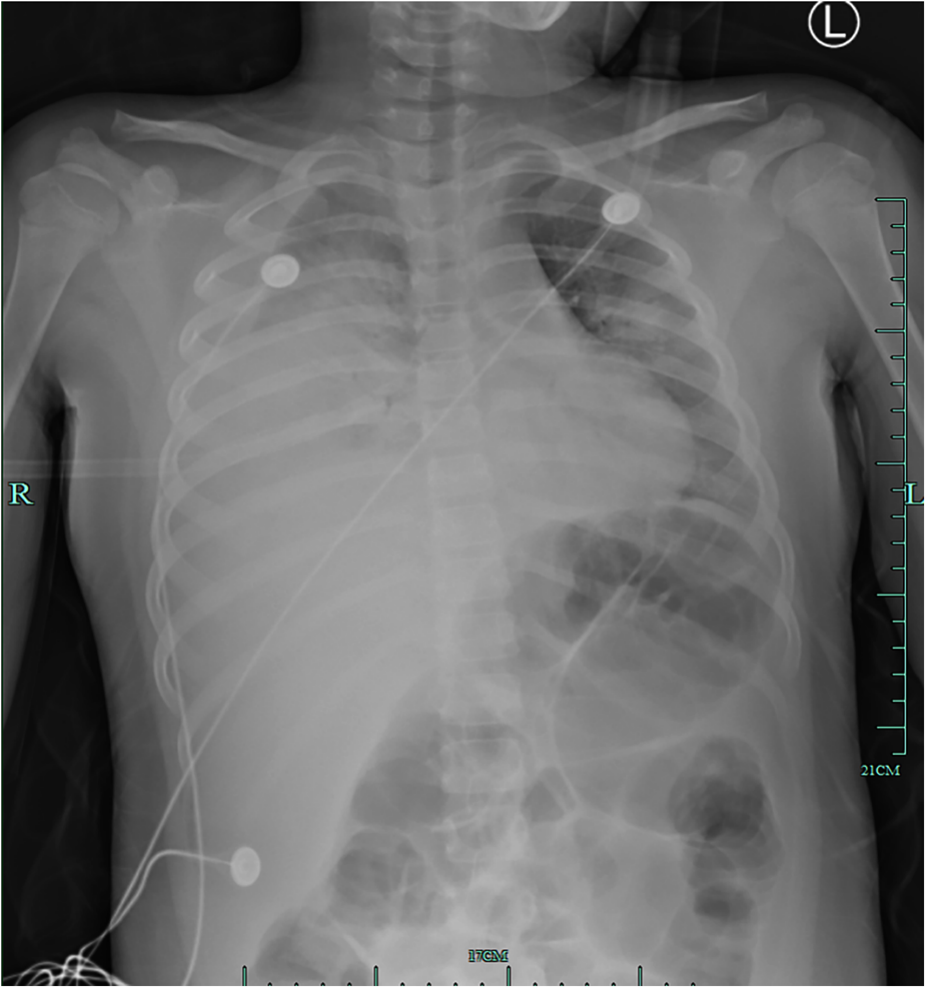
Initial chest radiograph on hospitalization day 1 showing diffuse opacification on the left lung, atelectasis in the right lung, and a large effusion in the right pleural cavity.

**Figure 2:**
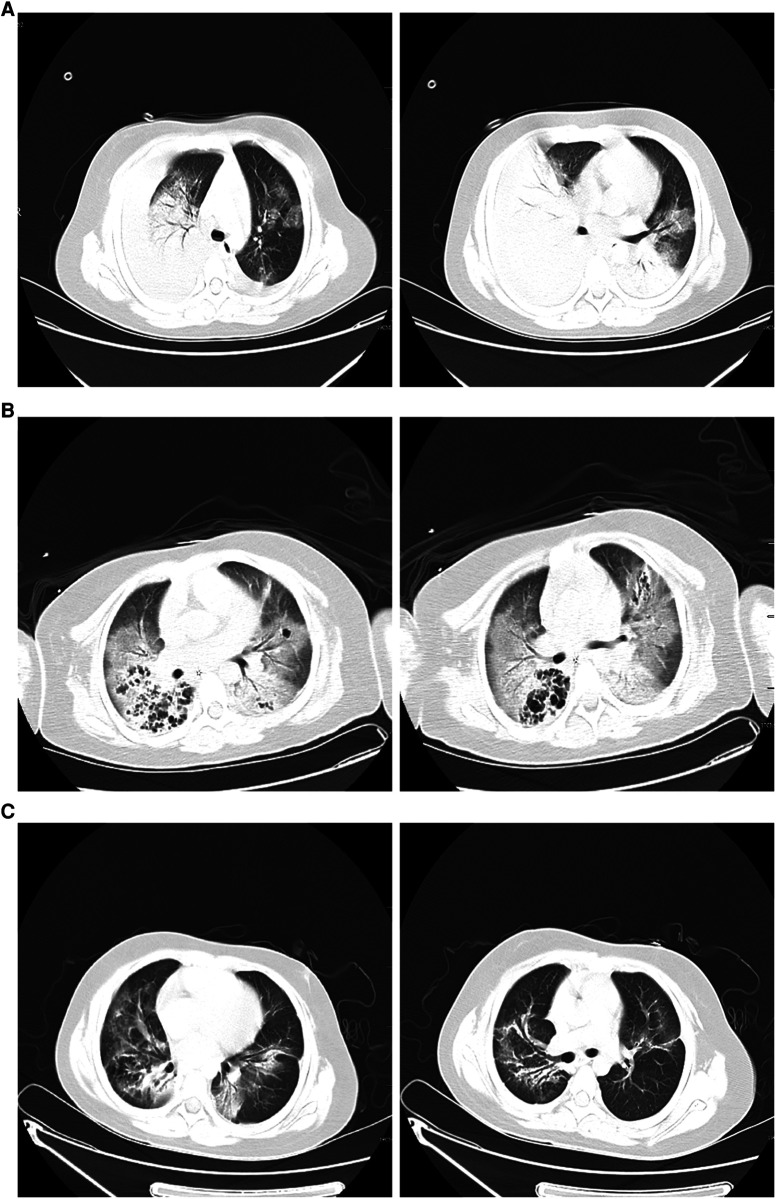
(**A**) Chest computed tomography scan on hospitalization day 1 showing double lung infection and right lung consolidation density; multiple lymph nodes in mediastinum the mediastinum show that the area is slightly larger; bilateral pleural effusion. (**B**) After 8 days of therapy, a computed tomography scan of the chest shows exudate changes in both lungs, partial consolidation in the right lung, similar lesions as initially observed, interstitial changes in the right lower lung with multiple bronchial cystic changes, and reactive lymphadenopathy. (**C**) Forty-five days after hospital discharge, the pulmonary lesions on computed tomography were significantly absorbed compared to earlier.

An 8-year-old girl presented to a hospital pediatric emergency department with a 1-week history of cough, fever for 4 days, and dyspnea for 5 h, without any response to oral cephalosporins. When she was admitted to the hospital, she had shortness of breath, 50 beats per minute, fever, 38.3 °C, tachycardia, 154 beats per minute, and hypoxemia, 72% in ambient air. Physical examination revealed moist rales in the left lung and diminished breath sounds over the right lung. Leukocyte count was 19 × 10^9^/L, indicating leukocytosis of mainly neutrophils, and C-reactive protein level was 0.2 mg/dl. Blood biochemical analysis showed that the level of alanine aminotransferase was 516 U/L, lactate dehydrogenase was 2113 U/L, and sodium ions was 122 mmol/L. Chest x-ray photograph showed diffuse opacification on the left lung, Atelectasis was observed in the right lung with a large effusion in the right pleural cavity (Figure 1). She received a combination of antibiotics (azithromycin and ceftriaxone) to treat severe pneumonia and was transferred to the pediatric intensive care unit (PICU) for further treatment. There, she was given high-flow warm humidified oxygen through the nose. A computed tomography (CT) scan of the chest showed bilateral lung infection and right lung consolidation density; Multiplelymph nodes in the mediastinum show that the area is slightly larger; Bilateral pleural effusion (Figure 2A). Ultrasonic examination also showed a large amount of pleural effusion in the right lung. Lower chest puncture drainage was performed under ultrasound guidance, and antibiotic therapy was escalated to cefoperazone sodium, sulbactam sodium, and azithromycin. Analysis of pleural effusion showed that a protein level of 38.2 g/L, glucose of 6.57 mmol/L, lactate dehydrogenase of 3082 U/L, and mononuclear phagocyte predominance was observed. Negative bacterial culture results for blood, endotracheal aspirate and pleural effusion. Antigen testing for respiratory pathogens (including adenovirus, respiratory syncytial virus, influenza virus and EB virus) is suggestive of negative results. Polymerase chain reaction (PCR) showed that MP was positive in endotracheal aspirate and pleural effusion. MP-specific IgM and IgG antibodies from blood showed negative results. Acquired mutations on the ribosomal macrolide target were negative, suggesting that it was not a macrolide-resistant strain. Further medical history obtained from family members did not indicate any risk factors for tuberculosis; however, a gamma release assay was also sent for analysis. On the first day of admission, patient was found to have abnormal coagulation function, and bleeding spots appear on the skin the next day. In the coagulation profile, the international normalized ratio was 1.67, with a partial thromboplastin time of 31.5 s and a prothrombin time of 17.5 s. Thrombin time was 60.0 s. The fibrinogen function K value was 18.1 min, the fibrinogen and platelet function (angle) were 27.9, the platelet function was 21.2 mm, and the comprehensive index of coagulation function was −14.5. In order to exclude hematologic diseases, coagulation factors and plasma correction tests were added, and fresh frozen plasma and fibrinogen infusions were given, and the coagulation function was slightly improved. After 48 h, she remained febrile with worsening tachypnea and hypoxic respiratory failure, hemoptysis, and required intubation and ventilation. Blood was visible in the airway, and bronchoscopy and bronchoalveolar lavage revealed DAH. Therefore, a diagnosis of severe MP in conjunction with DAH, severe pediatric acute respiratory distress syndrome (ARDS), and acute hypoxic respiratory failure was established. Human blood immunoglobulin, epinephrine endotracheal instillation, and intravenous application of glucocorticoids were administered.

Because of ongoing hypoxia, she was cannulated onto veno-venous extracorporeal membrane oxygenation (VV-ECMO) on hospitalization day 3. The results of pleural fluid and blood tests for metagenomic next-generation sequencing (mNGS) suggested MP infection, with no other viral or bacterial infections. She was screened for rheumatological conditions, tuberculosis, autoimmune diseases, and tumour. Results did not suggest these situations.The patient stayed on ECMO for 5 d; Due to the significant prolongation of PT and APTT, anticoagulation was given without heparin or low-dose heparin regimen, and the anticoagulation target was gradually adjusted on the basis of fresh frozen plasma supplementation to prevent thrombosis in the extracorporeal membrane lung and pipeline. Laying in the prone position for for 10 h + per day during this period improved oxygenation. Ultrasound was used to assess lung exudation. The patient was ventilated for ten days. During this period, she received methylprednisolone 1–2 mg/kg/d and a 14-day tapering regimen for acute respiratory distress syndrome and azithromycin was given intravenously, for inflammation, three times a week. Simultaneously, further testing revealed that the patient's humoral and cellular immune function was disorder, the TBNK lymphocyte subsets were suggested that CD3 799 × 10^6^/L(normal 960–3640), CD4 344 × 10^6^/L(normal 550–2190), CD8 440 × 10^6^/L(normal 260–1380), CD4/CD8 0.78(normal 0.72–2.88),natural killer cell 60 × 10^6^/L(normal 80–680); Cytokine detection suggested that IL-6 1.33 pg/ml(normal 0–20), IL-10 27.56 pg/ml(normal 0–5.9); Immunoglobulins and complements were shown that IgA0.37 g/L(normal 0.52–2.16), IgM0.46 g/L(normal 6.09–12.85), C3 0.25 g/L(normal 0.79–1.52), C4 0.09 g/L(normal 0.12–0.36). Hemophagocytic lymphohistiocytosis was negative. Mycoplasma DNA continued to test positive on repeat workups.

On the 9th day, the patient had low-grade fever; sputum culture, blood mNGS testing, and G test suggested *Candida albicans* infection, and fluconazole antifungal therapy was administered for 10 d. Other infectious organism was not found in repeated respiratory tract, blood cultures andpleural cultures. After 8 d of therapy, CT of the chest revealed exudative changes in both lungs, partial consolidation in the right lung, similar to the range of lesions as before, interstitial changes in the right lower lung with multiple bronchial cystic changes, and reactive lymphadenopathy (Figure 2B). After 12 d of therapy, the patient clinically improved and became afebrile. Ventilator use was discontinued, and she was discharged from the hospital 15 d after extubation without supplemental oxygen. Pulmonary CT review 45 days after discharge indicated that the lesion was significantly absorbed compared to the lesion in the previous CT scan (Figure 2C).

Written informed consent to participate in this study was provided by the participant's legal guardian/next of kin. We obtained informed written consent from the patient's parent authorizing the publication of this clinical case and images.

## Discussion

MP is one of the most common causes of community-acquired pneumonia in children. MP pneumonia is typically self-limiting and rarely requires mechanical ventilation or hospitalization. It rarely causes life-threatening diseases in children with normal immune ability but can be very severe (4–6). Severe mycoplasma infections are rare, and only 0.5%–2% of cases have a fulminant course (6). Key clinical and radiological findings of fulminant MP infection involve acute respiratory failure with diffuse consolidation or an abnormal interstitial pattern on chest radiograph. Izumikawa observed pleural effusion in 13.5% of cases with lymphocyte predominance (6). Herein, we describe a severe life-threatening case of infection with a macrolide-sensitive MP strain. This patient's condition progressed rapidly. We found this case remarkable because of (1) the approach to diagnosing MP, with pleural effusion samples and PCR/mNGS of the lower airways; (2) the presenting features of large pulmonary lesions and DAH, both rare in MP; and (3) the management strategies used, including methylprednisolone, prone position and VV-ECMO.

To our knowledge, DAH associated with MP is very rare; there are only five other published cases in adults, and there have been no reports in children (7–11). DAH is a life-threatening medical emergency with nonspecific symptoms and can result in respiratory failure and death. It is a pulmonary hemorrhagic syndrome caused by a disruption of the alveolar-capillary basement membrane due to injury to the microcirculation of the lungs, such as venules, alveolar capillaries, and arterioles (12). Hospital mortality rates are reported to be 20%–100% (1). Early diagnosis and treatment are critical to survival. The symptoms of DAH can present at any age, either with a previously diagnosed condition or as the first sign of a pre-existing condition. The most prevalent symptoms are hemoptysis, anemia, and new lung infiltrates. DAH is confirmed by hemorrhagic bronchoscopic bronchoalveolar lavage (BAL) on successive samples. The treatment is determined by the cause of the hemorrhage. The most prevalent cause of DAH is vasculitis, which is followed by thrombocytopenia, autoimmune illnesses, post-autologous stem cell transplantation, coagulation disorders, medications, and infections (2). The most common pathogens of infection are cytomegalovirus, Legionella, influenza A (H1N1), dengue, and staphylococcus. Reports of DHA due to Mycoplasma infection are rare and mostly affect individuals with weak immunity (3). We encountered an immunocompetent child with acute hypoxic respiratory failure due to MP-associated DAH requiring mechanical ventilation and VV-ECMO. Generally, pulmonary infections are infrequently related to DAH, but if left untreated, the condition has a high mortality rate and therefore should always be considered in the initial diagnosis (7).

Clinical characteristics and CT findings are not specific for detecting and diagnosing severe MP pneumonia initially. To minimize exacerbation of symptoms, early and exact laboratory diagnosis of MP infection is critical. Previous approaches, such as serological tests and mycoplasma culture, which might take a few weeks for the results to be available, are no longer feasible. The most sensitive and specific detection is real-time PCR of respiratory tract or nasopharynx samples, this is the preferred type of sample in most clinical settings (13–15). Early mNGS for MP utilizing pleural effusion fluid or serological fluid may be a viable option in individuals with dry cough without phlegm and when obtaining a lower respiratory tract sample is problematic. Studies have shown that PCR/mNGS of pleural effusion samples and lower respiratory tract can provide an early and rapid diagnosis of severe MP pneumonia with ARDS (16). In our case, in addition to collecting sputum, BAL fluid or endotracheal aspiration for PCR of MP, we also performed macro gene second-generation sequencing to detect *Mycoplasma* and other pathogens; the results are fast and sensitive. Related studies have found that mNGS technology can identify pathogens in patients with severe pneumonia at an early stage and guide the use of antimicrobials, thereby significantly reducing the 28- and 90-day case fatality rates of children with severe pneumonia (17). However, mNGS also has certain limitations as there is no international unified standard that can be used as a reference before the etiology is found.

DAH management entails an etiological diagnosis, vigorous supportive care, and treatment of any underlying systemic disease. In children with MP, macrolides are the treatment of choice; nonetheless, there are rising worries about the development of resistance (18). The only resistance mechanism reported is acquired mutations on the ribosomal macrolide target (19). Resistance may be present in up to a quarter of patients in Europe and the United States, whereas resistance may be present in more than 90% of patients in Japan and China (20). Our case, although fulminant, involved a non-resistant MP strain. Other underlying systemic diseases were excluded. Initially, the child received azithromycin combined with cephalosporin, low-dose glucocorticoid as an anti-inflammatory, and gamma globulin supportive therapy. A randomised controlled trial was conducted by Li et al. They randomly assigned children with refractory Mycoplasma pneumoniae pneumonia to group A [intravenous azithromycin (IA) + methylprednisolone (2 mg/kg/day for 3 days)], group B [IA + intravenous immunoglobulin (400 mg/kg/day for 3 days)] or group C (IA alone). After 7 days of treatment, the combined treatment groups A and B showed higher rates of infiltrative absorption, resolution of atelectasis and disappearance of pleural fluid compared to the control group C (21). Our report is similar to theirs, on the 5th day of admission, the child's body temperature returned to normal, and the pleural effusion was gradually absorbed. However, Zhang et al. reported that some children with severe or refractory MP required high-dose methylprednisolone (10–30 mg/kg/d) treatment with rapid recovery of pleural effusion and clinical symptoms (22).

Surveys have shown that some patients with MP develop severe hypoxic respiratory failure or severe ARDS and require ECMO rescue therapy (16). In the previous trial, the overall survival rate of mycoplasma pneumonia needing ECMO was 72.7%, suggesting that ECMO may be used to treat severe MP infection safely and successfully. In summary, the diagnosis of MP should be explored and studied in cases of DAH, severe ARDS, or other substantial cardiopulmonary illnesses, and unusual antimicrobial therapies should be undertaken. Because of the high percentages of survival shown in the literature and the Extracorporeal Life Support Organization database, ECMO should be considered in severe cases of MP (23). During ECMO, our patient was ventilated in the prone position (PP), and oxygenation improved significantly. Despite VV-ECMO support, patients with severe hypoxemia can consider PP. In patients with severe ARDS and COVID-19, PP under VV-ECMO improved the respiratory mechanics and oxygenation parameters, and the effects of PP on respiratory mechanics still existed after supine position reduction (24). There are more reports of ECMO combined with prone ventilation in adults, but there is a lack of data in children, we need a lot more clinical data on children to support it.

Studies have shown that lung lesions exceed two thirds of the lung volume, and the incidence of necrosis and embolism increases. Necrotizing pneumonia caused by MP is severe, although self-limiting and reversible. Good outcomes can be achieved with appropriate management (25). In our case, the child's CT suggested bronchial cystic changes without embolization. After treatment, the pulmonary lesions were obviously absorbed in the subsequent follow-up, and the child did not require oxygen support when discharged.

In conclusion, large lung lesions associated with MP and the presenting features of DAH are very rare and a high level of vigilance should be maintained for rapid outbreaks of this disease The rapid and early diagnosis of severe MP pneumonia can be realized by PCR/mNGS of samples from pleural effusions or lower respiratory tract. Diagnosis of DAH and other associated infections can be achieved by bronchoscopy and BAL. The main therapeutic measures include anti-infection and supportive measures such as mechanical ventilation, but as in our case, success in prone position and VV-ECMO was life-saving.

## Data Availability

The original contributions presented in the study are included in the article/Supplementary Material, further inquiries can be directed to the corresponding author/s.
